# Overcoming the Barrier of the Respiratory Epithelium during Canine Distemper Virus Infection

**DOI:** 10.1128/mbio.03043-21

**Published:** 2022-01-18

**Authors:** Dai-Lun Shin, Elisa Chludzinski, Nai-Huei Wu, Ju-Yi Peng, Malgorzata Ciurkiewicz, Bevan Sawatsky, Christian K. Pfaller, Christine Baechlein, Veronika von Messling, Ludwig Haas, Andreas Beineke, Georg Herrler

**Affiliations:** a Institute of Virology, University of Veterinary Medicine Hannover, Hannover, Germany; b Institute for Pathology, University of Veterinary Medicine Hannover, Hannover, Germany; c Department of Veterinary Medicine, National Taiwan University, Taiwan; d Division of Veterinary Medicine, Paul-Ehrlich-Institut, Federal Institute for Vaccines and Biomedicines, Langen, Germany; e German Center for Infection Research, TTU Emerging Infections, Langen, Germany; Columbia University Medical College

**Keywords:** canine distemper virus, air-liquid interface, cell-to-cell transmission

## Abstract

Canine distemper virus (CDV) is a highly contagious pathogen and is known to enter the host via the respiratory tract and disseminate to various organs. Current hypotheses speculate that CDV uses the homologous cellular receptors of measles virus (MeV), SLAM and nectin-4, to initiate the infection process. For validation, here, we established the well-differentiated air-liquid interface (ALI) culture model from primary canine tracheal airway epithelial cells. By applying the green fluorescent protein (GFP)-expressing CDV vaccine strain and recombinant wild-type viruses, we show that cell-free virus infects the airway epithelium mainly via the paracellular route and only after prior disruption of tight junctions by pretreatment with EGTA; this infection was related to nectin-4 but not to SLAM. Remarkably, when CDV-preinfected DH82 cells were cocultured on the basolateral side of canine ALI cultures grown on filter supports with a 1.0-μm pore size, cell-associated CDV could be transmitted via cell-to-cell contact from immunocytes to airway epithelial cultures. Finally, we observed that canine ALI cultures formed syncytia and started to release cell-free infectious viral particles from the apical surface following treatment with an inhibitor of the JAK/STAT signaling pathway (ruxolitinib). Our findings show that CDV can overcome the epithelial barrier through different strategies, including infection via immunocyte-mediated transmission and direct infection via the paracellular route when tight junctions are disrupted. Our established model can be adapted to other animals for studying the transmission routes and the pathogenicity of other morbilliviruses.

## INTRODUCTION

Canine distemper virus (CDV), a member of the family Paramyxoviridae (genus Morbillivirus), is an enveloped virus with a nonsegmented RNA genome of negative polarity. Representatives of the morbillivirus genus are characterized by (i) high contagiousness, (ii) spread through respiratory droplets, (iii) pronounced immunosuppression, (iv) large outbreaks in unprotected populations, associated with high morbidity or mortality, and (v) induction of a lifelong immunity in surviving hosts. The prototype of morbilliviruses is measles virus (MeV). Its pathogenesis is challenging to study, since humans are the only natural hosts, and nonhuman primates are the only suitable experimental animal models. In contrast to the narrow host range of MeV, CDV infects a wide range of carnivores, including dogs, foxes, seals, lions, and hyenas (1–5). Studies using ferrets and nonhuman primates show that after viruses have entered the host within droplets or aerosols, they invade cells by fusing their viral membranes with cell membranes. In this stage, it is speculated that CDV—similarly to MeV—may overcome the epithelial barrier within infected residential immune cells, such as macrophages (6, 7). Later, virus proliferates in immune cells of the respiratory tract, predominantly in local macrophages. From there, the infection spreads to tonsils and bronchial lymph nodes, and subsequently to other components of the lymphatic system, such as the spleen, the thymus, and the lymph nodes (8). Moreover, the virus shows a pronounced neurotropism, and often causes fatal disease (9, 10).

Two cellular receptors for the virus have been identified so far. Signaling lymphocyte activation molecule, family member 1 (SLAMF1; also referred to as CD150) is expressed on B and T lymphocytes, dendritic cells, hematopoietic cells, and macrophages, which explains the pronounced tropism of this virus for immune cells (11, 12). The second receptor is nectin cell adhesion molecule 4 (nectin-4), a component of adherens junctions of epithelial cells (13, 14). A notable feature of morbilliviruses is their ability to infect cells of the respiratory tract, from which infectious virus is released before being transmitted to other hosts via aerosols or respiratory droplets. This makes the infection process related to respiratory epithelial cells a major topic for morbillivirus research. The concept of direct infection of airway epithelial cells by morbilliviruses in the early phase of infection has been modified in recent years, as the main receptor (SLAM) is completely absent on these cells, and the second receptor (nectin-4) has a basolateral localization and thus is not available for infection from the luminal side. In macaques, immune cells (macrophages and dendritic cells) of the alveolar region were identified as primarily infected cells (6). Based on this finding, it has been concluded, that morbilliviruses use immune cells as vehicles to overcome the barrier of the respiratory epithelium in the alveolar region (6, 15). However, it appears unlikely that a virus as highly contagious as CDV, which—upon inoculation at a low dose—can infect animals and induce lethal disease, initiates infection exclusively in the lower respiratory tract (7). It therefore remains to be clarified whether overcoming the epithelial barrier by cell-free virions of CDV is also possible in the upper respiratory tract or whether unknown adjunctive factors make the respiratory epithelium permeable to morbilliviruses.

To date, several *in vivo* studies have characterized the pathogenicity and the infection route of CDV. For instance, CDV-5804PeH has been reported as a suitable pathogen to trace infection in the ferret model (16). Other CDV strains, like recombinant Snyder Hill viruses, which express different fluorescent proteins, provide the possibility to study the real-time dissemination of CDV in ferrets (7). On the other hand, for elucidating molecular interactions and to reduce the usage of experimental animals, alternative models have been developed for characterizing the virulence of morbilliviruses. Recently, airway epithelial cultures have been applied to study the virulence of MeV *in vitro*. These cultures have differentiated on filter supports to specialized cells and show characteristic features of airway epithelial cells, including (i) a pseudostratified morphology, (ii) a strong barrier function, and (iii) a mucociliary clearance system comprising ciliated and mucus-producing cells (17).

The knowledge about the pathogenesis of canine distemper is still fragmentary. In particular, the route of CDV infection in the respiratory tract is poorly understood. Therefore, we explored how CDV overcomes the epithelial barrier of the respiratory tract and how the immune cells participate during this process. In the present study, we analyzed cell-associated virus infection and cell-free virus infection. In addition, the influence of the local immune system on the virus spreading was examined more closely.

## RESULTS

### CDV infects airway epithelial and subepithelial cells in naturally infected dogs.

Immunohistochemistry of lung tissues from naturally CDV-infected dogs (see Fig. S1A and B in the supplemental material) revealed the presence of CDV antigen within airway epithelial cells and subepithelial cells, which show a histiocytic morphology. These observations from naturally infected canine lungs slices support the view that CDV infects the airway epithelium and overcomes its barrier function.

10.1128/mBio.03043-21.1 FIG S1 Immunohistochemical staining of a canine lung following natural canine distemper virus (CDV) infection. Immunohistochemical staining against CDV nucleoprotein in lung tissue from a naturally CDV-infected dog (A and B). Airway epithelial cells (triangles) and subepithelial immunocytes (arrows) show positive labeling for CDV antigen. Bars, 50 μm (A), 20 μm (B). Download FIG S1, TIF file, 1.8 MB. Copyright © 2022 Shin et al. 2022 Shin et al. https://creativecommons.org/licenses/by/4.0/ This content is distributed under the terms of the Creative Commons Attribution 4.0 International license.

### Establishment of air-liquid interface cultures of primary tracheal epithelial cells.

ALI cultures are a powerful system to analyze virus replication in well-differentiated airway epithelial cells, since they mimic the microenvironment of the respiratory tract epithelium. In order to get a closer look at how CDV overcomes the barrier of the airway epithelium, we established a canine ALI culture system and used ferret ALI culture for experiments as well.

**FIG 1:**
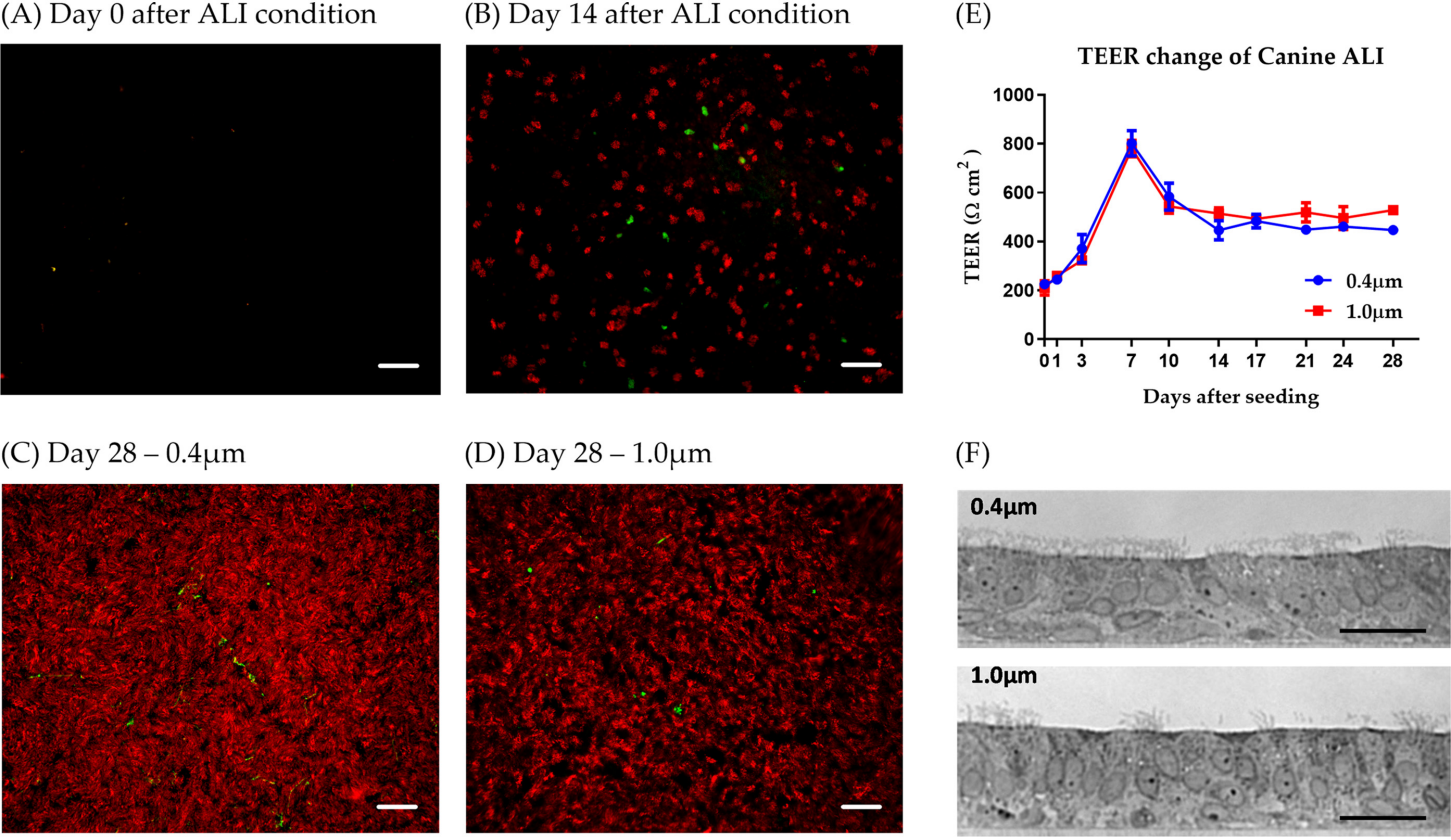
Characterization of primary canine tracheal epithelial cultures under air-liquid interface conditions. Primary canine tracheal epithelial cultures were grown on polyester filter supports with pore sizes of either 0.4 μm (A to C) or 1.0 μm (D). On day 4 after seeding onto the filter supports, the medium from the upper chamber was removed, and the air-liquid interface condition was maintained until 28 days after seeding. On days 0 (A), 14 (B), and 28 (C and D) after cells changed into ALI conditions, the canine ALI cultures were fixed with 3% paraformaldehyde (PFA), and the cellular markers were stained with specific antibodies (ciliated cells, β-tubulin, red; mucus-producing cell, mu5AC, green) to characterize the differentiation of the ALI cultures. On the day of analysis, four individual filters were used to measure the resistance value indicating the polarity of the epithelial cultures (E). Semithin sections of the epoxy resin-embedded canine ALI cultures were produced (F). Bars, 100 μm (A to D), 25 μm (F).

ALI cultures are based on primary epithelial cells from the canine trachea, seeded on filter supports with a pore size of either 0.4 μm or 1.0 μm. On day 4 postseeding, the canine ALI medium was removed from the upper chamber, and cells were maintained under air-liquid conditions for 28 days. On days 0 (start of air-liquid interface conditions), 14, and 28, filters were subjected to immunostaining to determine the state of cell differentiation. Antibodies directed against β-tubulin and mucin-5AC (mu5AC) were applied to visualize ciliated cells and mucus-producing cells, respectively. As shown in Fig. 1A and B, both cell types were already present after a culturing period of 14 days. No significant differences in cell morphology were observed between the canine ALI cultures grown with pore sizes of 0.4 μm or 1.0 μm (Fig. 1C and D).

The transepithelial electrical resistance (TEER) was determined to analyze the barrier function of the differentiated airway epithelium. As shown in Fig. 1E, resistance values peaked on day 7 of ALI conditions and remained stable until day 28. To characterize the morphology of the filter-grown airway epithelium, semithin sections were analyzed by microscopy, showing the appearance of a pseudostratified epithelium (Fig. 1F) characteristic for canine tracheal epithelium (Fig. S1).

### Canine distemper virus is transmitted from immune cells to epithelial cells.

The initial step of CDV infection *in vivo* is assumed to be the picking up of virions by immune cells within the respiratory tract. To determine whether CDV crosses the airway epithelium within immune cells, which may transmit the virus by cell-to-cell-fusion to either epithelial cells or subepithelial cells, an immunocyte-epithelial transwell coculture system was established. Here, we used CDV-preinfected DH82 cells to mimic macrophages, which contain cell-associated virus particles, and used canine ALI cultures to represent the airway epithelium for the following experiments.

**FIG 2:**
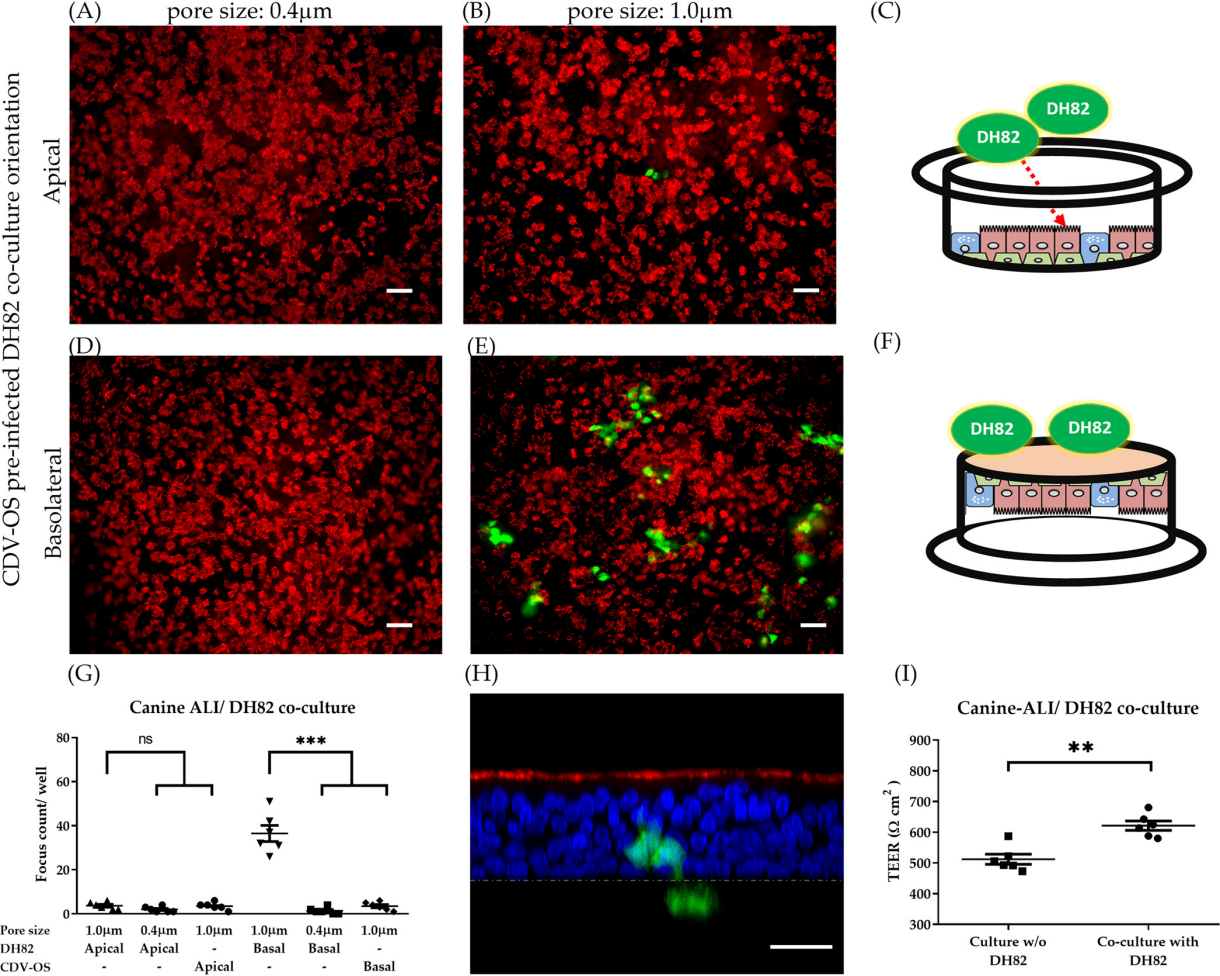
Cell-to-cell transmission of CDV between immunocytes and airway epithelial cells. Three days prior to coculture experiments, DH82 cells were infected with CDV-OS (MOI = 0.1). On the day of coculture, 200 CDV-OS-preinfected DH82 cells were added to the apical (A to C) or basolateral (D to F) orientations of the canine ALI cultures grown on filters with pore sizes of 0.4 μm (A and D) or 1.0 μm (B, E, and H). On day 3 after initiating the coculture, the DH82/canine ALI cultures were fixed with 3% PFA, and the ciliated cells were stained with Cy3-conjugated antibody against β-tubulin (red). The numbers of CDV-OS-related foci (green) were calculated and statistically analyzed (G). The Z-section of the DH82/canine ALI cocultured samples of Fig. 4E with a positive signal of CDV-OS was visualized by using Leica confocal microscope (H); The nuclei of canine ALI cultures were counterstained with DAPI (blue). The yellow dashed line indicates the location of the transwell membrane with a pore size of 1.0 μm. TEER changes after DH82 cells cocultured with canine ALI (0.1-μm filter) on day 1 post coculture (I). Bars, 50 μm (A, B, D, and E), 25 μm (H). Statistical analysis: one-way analysis of variance (ANOVA) with Tukey’s *post hoc* test; **, *P* < 0.01; ***, *P* < 0.001; ns, not significant.

Three days prior to immunocyte-epithelial cell coculturing, DH82 cells were infected with recombinant CDV based on the Onderstepoort vaccine strain (CDV-OS) with a multiplicity of infection (MOI) of 0.1. On the day of coculturing, infected DH82 cells were added to canine ALI cultures either to the phosphate-buffered saline (PBS)-washed apical chamber or to the basolateral surface. On day 3 postcoculturing, cells were fixed, and ciliated cells were stained by an antibody against β-tubulin (Fig. 2). Results show that canine ALI cultures grown on filter supports with a pore size of 0.4 μm were not infected by CDV, irrespective of the location of immune cells. However, when canine ALI cultures were grown on filter supports with a pore size of 1.0 μm, infected epithelial cells were readily detectable when preinfected DH82 cells were applied to the basolateral side of the filter. In contrast, an apical location of DH82 cells resulted only in a very low number of infected epithelial cells (Fig. 2G). Basolateral or apical application of cell-free CDV-OS virus to canine ALI cultures led to only low numbers of infected cells, irrespective whether the pore size was 0.4 or 1.0 μm (Fig. 2G). Despite removal of mucus by washing with PBS prior to coculturing, preinfected DH82 cells applied to the apical side of canine ALI cultures could not form stable cultures and were easily removed via the mucociliary clearance function of ALI cultures by day 3 (Fig. 2A and B). Vertical sections of cocultures analyzed by confocal fluorescence microscopy (Fig. 2H) revealed that preinfected DH82 cells (green, below the yellow dashed line) attached to the basolateral surface of the filters (yellow dashed line) and infected epithelial cells (green, above the dashed line) were present on the apical surface. The coculture of DH82 cells with canine ALI cultures slightly increased the TEER, probably due to the increased number of cells on the filter support (Fig. 2I). These observations illustrate that preinfected DH82 cells are able to transmit CDV to epithelial cells through a pore size of 1.0 μm.

### Opening of tight junctions renders well-differentiated airway epithelial cells susceptible to infection with cell-free canine distemper virus.

In the previous section, we analyzed cell-mediated infection. To address whether cell-free virus can also contribute to the infection of epithelial cells, we analyzed the direct infection of ALI cultures by CDV.

**FIG 3:**
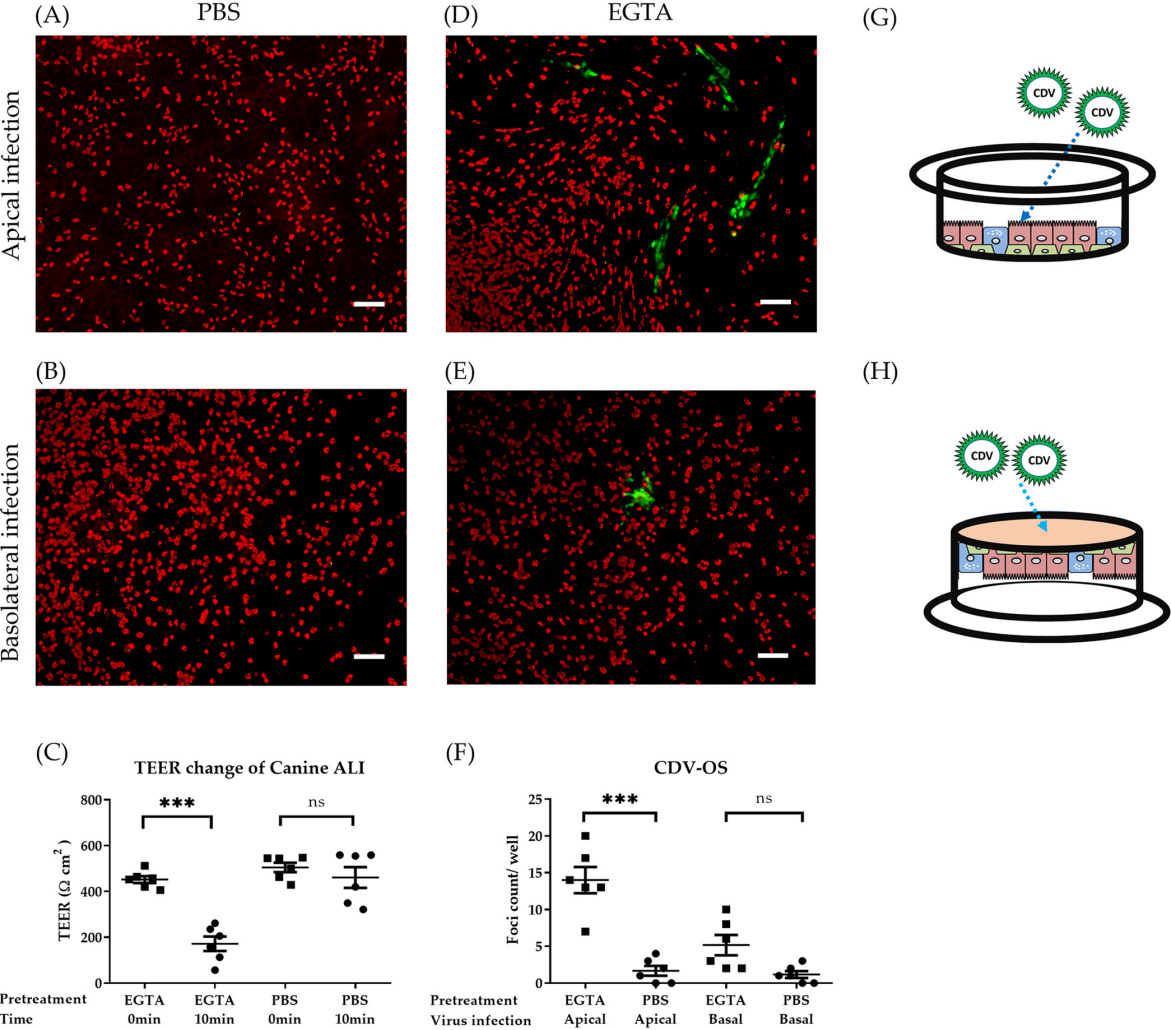
Disruption of tight junctions enables CDV-OS infection of well-differentiated canine ALI cultures. Canine ALI cultures grown on filter supports with 0.4-μm pore size were inoculated by CDV-OS (MOI = 0.5). Prior to cell-free virion inoculation, cells were pretreated with PBS containing magnesium-calcium or with 100 mM EGTA in PBS (without magnesium-calcium) on the apical and basolateral sides. Before and after the incubation time of 10 min, the TEER values of the treated ALI cultures were measured (C). Following this pretreatment, CDV-OS was inoculated on the apical surface or onto the basolateral surface in an upside-down position for 2 h. On day 3 postinoculation, the ALI cultures were fixed by 3% PFA, and the ciliated cells were stained with antibodies against β-tubulin. The CDV-OS eGFP signals were visualized under the Nikon fluorescence microscope (A, B, D, and E), and the numbers of virus foci (green) were calculated and statistically analyzed (F). Schemes describing apical inoculation (G) and basolateral cell-free virion inoculation method with upside-down position (H). Bar, 100 μm. Statistical analysis: one-way ANOVA with Tukey’s *post hoc* test; ***, *P* < 0.001; ns, not significant.

We inoculated canine ALI cultures by cell-free virions from either the apical or basolateral side. Viruses were first analyzed for their ability to infect Vero.dog-SLAMtag (VDS), Madin-Darby canine kidney (MDCK), and DH82 cell lines at an MOI of 0.1 (Fig. S2). On day 3 postinfection, cells were analyzed by immunofluorescence microscopy. The well-differentiated airway epithelial cultures were rather refractory to cell-free virion inoculation by CDV at an MOI of 0.5. As shown in Fig. 3A and B, no virus-infected cells were detected after either apical or basolateral inoculation. Additional infection experiments with canine parainfluenza virus type 5 were performed (MOI of 0.5), which showed that canine ALI cultures could be efficiently infected from both the apical and the basolateral side (Fig. S3). To analyze the effect of tight junctions on the susceptibility to infection, well-differentiated epithelial cultures were incubated with EGTA for 10 min. This treatment led to an opening of tight junctions as indicated by the drop in TEER values (Fig. 3C). The loss of transepithelial electrical resistance was paralleled by an increased susceptibility to CDV infection (Fig. 3D and E). Inoculation of cell-free virions from the basolateral side resulted in only a few CDV-infected cells (Fig. 3E). However, after inoculation from the apical surface, a substantial number of infected cells were detected (Fig. 3D), which was significantly increased compared to those in cultures not treated with EGTA (Fig. 3F). Moreover, staining of EGTA-pretreated canine ALI cultures revealed an increased exposure of nectin-4 on the cell surface (Fig. S4A to C). The inability of CDV to infect the ALI cultures from the basolateral side might be related to insufficient nectin-4 expression, as discussed below.

10.1128/mBio.03043-21.2 FIG S2 Syncytial formation of different cells after infection by CDV and receptor-blind variants. Vero.dog-SLAMtag (VDS), MDCK, and DH82 cells were infected by recombinant CDV Onderstepoort (CDV-OS), CDV-5804PeH, and receptor-blind variants (SLAM blind and nectin-4 blind) at a multiplicity of infection (MOI) of 0.1. On days 2 (VDS) and 4 (MDCK and DH82) postinfection, the virus infection was visualized by detecting the enhanced green fluorescent protein (eGFP) fluorescence signal expressed by the viruses (green color in dark). The cytopathic effects of syncytium formation were observed by light field microscopy (phase). Bar, 100 μm. Download FIG S2, TIF file, 2.6 MB. Copyright © 2022 Shin et al. 2022 Shin et al. https://creativecommons.org/licenses/by/4.0/ This content is distributed under the terms of the Creative Commons Attribution 4.0 International license.

10.1128/mBio.03043-21.3 FIG S3 Canine parainfluenza virus infection of canine air-liquid interface (ALI) cultures. Well-differentiated canine ALI cultures were inoculated with canine parainfluenza virus type V (cPIV5) from the apical (A) or basolateral (B) side (MOI = 0.5). On day 3 postinoculation, the cultures were fixed by 3% paraformaldehyde (PFA), and cPIV5 antigen (green) was visualized by staining with rabbit anti-V5 tag antibody (Cell Signaling Technology) and an Alexa Fluor 488-conjugated secondary antibody (Invitrogen). The ciliated cells (red) were stained with Cy3-conjugated antibody against β-tubulin. The results indicate that cPIV5 is able to infect canine ALI cultures from both the apical and the basolateral side. Bar, 100 μm. Download FIG S3, TIF file, 2.7 MB. Copyright © 2022 Shin et al. 2022 Shin et al. https://creativecommons.org/licenses/by/4.0/ This content is distributed under the terms of the Creative Commons Attribution 4.0 International license.

10.1128/mBio.03043-21.4 FIG S4 Increase of nectin-4 exposure after EGTA pretreatment of canine ALI cultures. Canine ALI cultures were treated with EGTA or phosphate-buffered saline (PBS) for 10 minutes prior to the fixation (six filters per group). (A and B) After the cells had been fixed by 3% PFA, nectin-4 (red) was stained by a rabbit antibody against nectin-4 protein (Elabscience, USA); Alexa Fluor 568-conjugated goat-anti-rabbit antibody (Invitrogen) was used as a secondary antibody. (C) Percentage of nectin-4-positive pixels per field was calculated using ImageJ software. (D) Transepithelial electrical resistance (TEER) of canine ALI culture changes after PBS washes; nonwashed ALI culture contains a mucus layer. The results showed that after EGTA treatment, the percentage of visible nectin-4 was significantly increased compared to that in the PBS-treated group. Bar, 100 μm. Statistical analysis: Mann-Whitney U test; **, *P* < 0.01. FIG S4, TIF file, 1.8 MB Copyright © 2022 Shin et al. 2022 Shin et al. https://creativecommons.org/licenses/by/4.0/ This content is distributed under the terms of the Creative Commons Attribution 4.0 International license.

### Importance of nectin-4 for infection of airway epithelial cells by canine distemper virus.

**FIG 4:**
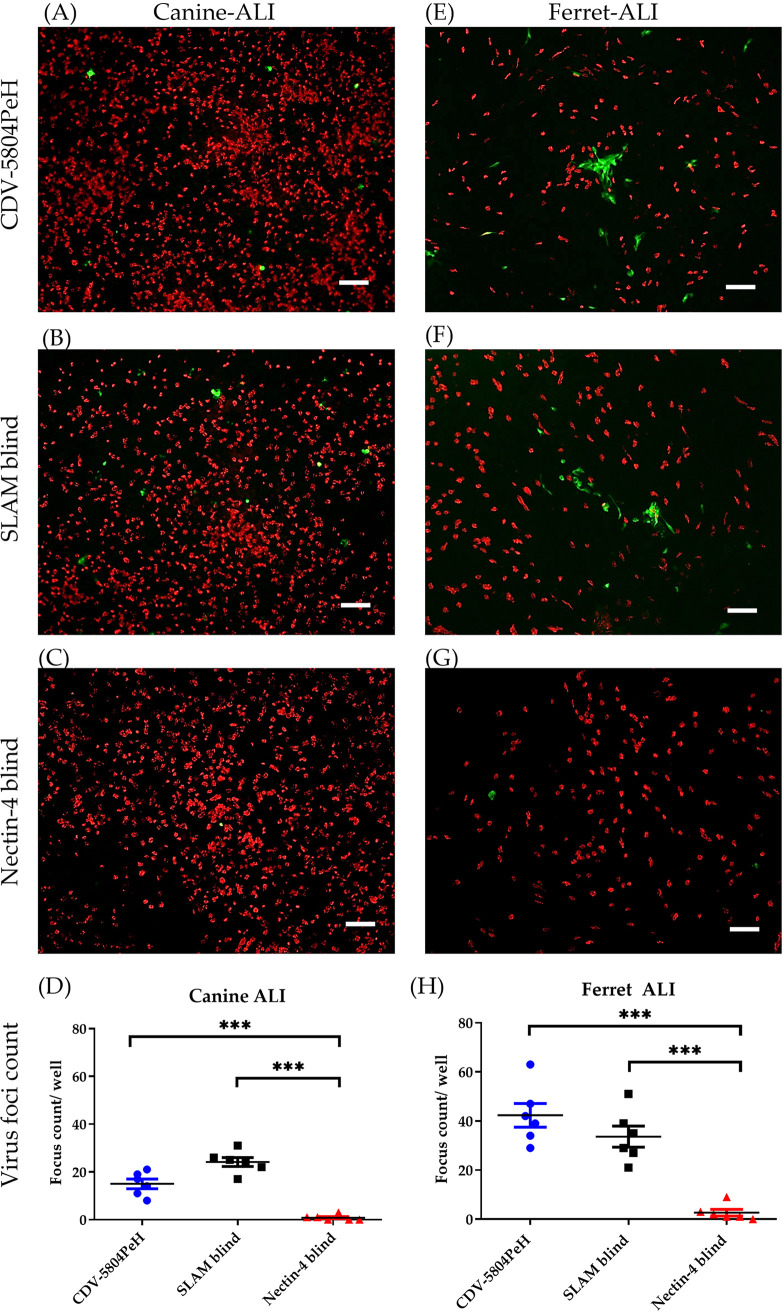
The paracellular route for CDV to enter the airway epithelial cells is governed by nectin-4. Ferret-adapted recombinant CDV-5804PeH and its mutants (SLAM-blind and nectin-4 blind viruses) were used to inoculate canine and ferret ALI cultures. After EGTA pretreatment for 10 min, the dog (A to D) and ferret (E to H) ALI cultures were inoculated with the recombinant viruses (MOI = 0.5) from the apical side as described above. On day 3 postinoculation, the ALI cultures were fixed by 3% PFA and the ciliated cells (β-actin, red) and signals for viral replication (eGFP, green) were visualized using Nikon fluorescence microscope (canine ALI, panels A to C; ferret ALI, panels E to G), and the numbers of virus foci (green) were calculated and statistically analyzed (canine ALI, panel D; ferret ALI, panel H). Bar, 100 μm. Statistical analysis: one-way ANOVA with Tukey’s *post hoc* test; ***, *P* < 0.001.

MeV uses nectin-4 as a receptor to infect epithelial cultures from the basolateral side. To determine whether cell-free CDV uses the same receptor for epithelial infection, canine ALI cultures were inoculated by recombinant SLAM-blind virus and nectin-4-blind virus after EGTA pretreatment. These recombinant viruses are derived from CDV-5804PeH. Following the protocol described above, well-differentiated airway epithelial cultures were pretreated with EGTA and respectively inoculated by recombinant viruses or CDV-5804PeH at an MOI of 0.5. On day 3 postinoculation, ALI cultures were double immunostained for the presence of ciliated cells (red) and virus-infected cells (green). As shown in Fig. 4A to D, EGTA-pretreated canine ALI cultures were susceptible to infection by both CDV-5804PeH and SLAM-blind virus to a similar degree. In contrast, epithelial inoculation by nectin-4-blind virus was significantly decreased compared to that by CDV-5804PeH and SLAM-blind virus, respectively.

Since CDV-5804PeH had been previously adapted to ferrets, ferret ALI cultures were used to validate the findings obtained by canine ALI cultures. Using the same method, the EGTA-pretreated ferret ALI cultures were inoculated by recombinant CDVs. The results obtained with ferret ALI cultures were similar to those determined for canine ALI cultures (Fig. 4E to H), i.e., infection was detectable only with parental virus and SLAM-blind virus but not with nectin-4-blind virus. A notable difference between these two culture types was that virus foci of infected cells were larger in ferret ALI cultures (about 120 μm) than those in infected canine ALI cultures (about 20 μm). The results described here demonstrate that cell-free CDV infection of well-differentiated airway epithelial cultures is mediated by nectin-4.

### The JAK/STAT pathway regulates canine distemper virus-induced syncytium formation and virus release from airway epithelial cells.

Data indicate that CDV is able to infect the airway epithelium paracellularly and through cell-to-cell transmission. Since CDV can spread via aerosols to other individuals, virus release from the infected airway epithelium and the impact of local immunosuppression was investigated.

In the experiments shown in Fig. 2 to 4, CDV infection was characterized by analyzing the presence of infected differentiated airway cells. To get information about the release of CDV infectious particles, the apical and basolateral supernatants of the respective filter-grown cultures were collected and analyzed. Aliquots were centrifuged at 3,500 × *g* for 1 min to separate cell-free and cell-associated fractions to detect infectious CDV particles. The samples were directly applied to VDS cells and incubated for 6 days to allow syncytium formation or the development of cytopathic effects. When canine ALI cultures had been inoculated with cell-free CDV (Fig. 3 and 4), the virus recovered from these cells was mainly detected in the cell-associated fraction, but not in the cell-free fraction (Table 1). Interestingly, when canine ALI cells were cocultured with infected DH82 cells on the basolateral side, the supernatant collected from the bottom chamber was able to induce the formation of syncytia in VDS cells, which may be due to the release of cell-free virus from the remaining adherent DH82 cells.

**TABLE 1:** Numbers of canine ALI cultures positive for cell-free or cell-associated infectious virus on day 3 after CDV-OS inoculation

Pretreatment	Inoculation side	Sampling	No. of positive cultures/total no. of cultures^*a*^	
			Cell-free virus	Cell-associated virus
PBS	Apical	Apical surface	0/6	0/6
		Basolateral medium	0/6	ND
	Basolateral	Apical surface	0/6	0/6
		Basolateral medium	0/6	ND
EGTA	Apical	Apical surface	1/8	8/8
		Basolateral medium	0/8	ND
	Basolateral	Apical surface	0/8	2/8
		Basolateral medium	0/8	ND

a Numbers indicate cytopathic effect (CPE) formation on VDS (no. positive/total no. of supernatants or cell pellets) of the inocula from different CDV-OS-infected groups. A total of 28 filters from the two different animals were used to determine the virus release after phosphate-buffered saline (PBS) or EGTA pretreatment in canine ALI cultures. ND, no data were available.

**FIG 5:**
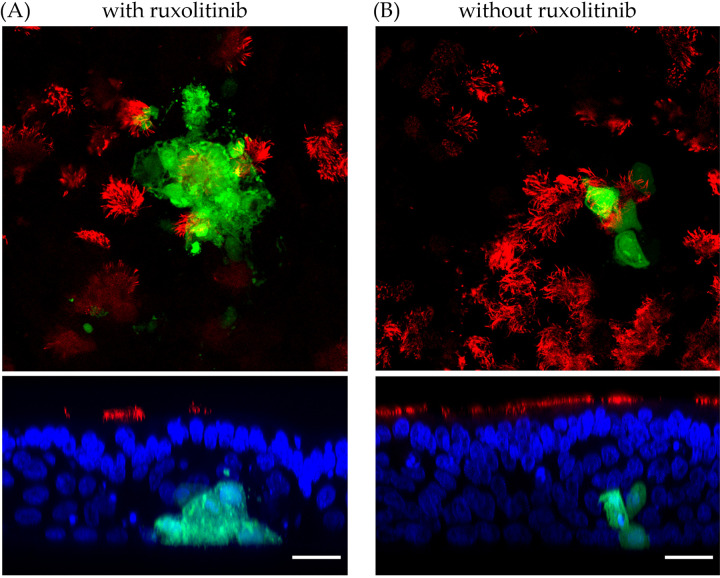
Inhibition of interferon signaling pathway affects the syncytium formation in the CDV-OS-infected ALI cultures. The potent JAK/STAT signaling pathway inhibitor ruxolitinib (2.5 μM) was supplied in the canine ALI medium 3 days prior to DH82/canine ALI coculture and during the experiments. After 3 days of coculturing CDV-OS-preinfected DH82 and canine ALI cultures, the samples were fixed by 3% PFA, and the ciliated cells were stained with Cy3-conjugated antibody against β-tubulin. The CDV-OS-infected cells showed a green color under the Leica SP5 confocal fluorescence microscope. (Upper) X-Y view of ruxolitinib-treated (A) and mock-treated (B) canine ALI culture groups; (lower) Z-section view of the CDV-OS-positive foci. The nuclei of the cells were counterstained with DAPI, shown in blue. Bar, 20 μm.

Morbilliviruses have been described to interfere with the JAK/STAT signaling pathway of the innate immune response (18). To mimic the inhibitory effect of the innate immune system within lung tissue of CDV-infected dogs, canine ALI cultures were treated with the STAT inhibitor ruxolitinib. Canine ALI cocultured with CDV-infected-DH82 cells from the basolateral side showed syncytium formation of infected epithelial cells after treatment with ruxolitinib (Fig. 5A), while the nontreated group showed no cytopathic effect in infected epithelial cells (Fig. 5B and Fig. 4H). In addition, when DH82/canine ALI cocultures were treated with ruxolitinib, infectious virus particles were detected in the apical inoculum (Table 2). The results described here with CDV-OS infection indicate that inhibition of the JAK/STAT signaling pathway is crucial for syncytium formation and virus release in well-differentiated airway epithelial cells.

**TABLE 2:** Numbers of DH82/canine ALI cocultures positive for release of infectious virus particles on day 6 post coculturing

DH82 coculture orientation	Sampling	No. of positive cultures/total no. of cultures^*a*^	
		Without ruxolitinib	With ruxolitinib
Apical	Apical surface	1/12	0/12
Apical	Basolateral medium	0/12	0/12
Basolateral	Apical surface	1/12	11/12
Basolateral	Basolateral medium	12/12	12/12

a Numbers indicate CPE formation on VDS cells (no. positive/total no. of inocula) from different coculture groups. Twelve filters from the three different animals were used to perform the DH82/canine ALI coculture and received ruxolitinib or mock treatment.

## DISCUSSION

CDV is known to be highly contagious in different carnivore species, including dogs, foxes, ferrets, seals, lions, and hyenas (10, 19, 20) and has been used to study the behavior of MeV infection *in vivo* using ferret models (21). The process of MeV infection comprises three major steps. During the early stage of MeV infection, macrophages in the alveolar lumen or dendritic cells beneath the basolateral part of the airway epithelium take the virus up from aerosols (6). Subsequently, viremia and systemic dissemination of the virus occurs (22). During the late stage, infected immunocytes transport MeV back to infect the airway epithelium and infectious virus particles are released into the airway lumen and transmitted to other hosts via aerosols (23). CDV has been used widely as a surrogate pathogen to study the pathogenicity of MeV *in vivo*; however, the current knowledge about transmission and virulence of CDV relies primarily on ferret models (24). Although there are several reports that support this hypothesis, validation of the stage in which morbilliviruses overcome the epithelial barrier is still lacking.

Several studies have used well-differentiated human airway epithelial cell cultures to investigate the entry route of MeV (25, 26), and airway epithelial cell cultures from macaques have been used to compare cell entry mechanisms between CDV and MeV (27). Cell-free MeV is able to infect human epithelial cells from the basolateral side (17, 28). A recent study showed that well-differentiated airway epithelial cells are actually highly susceptible to MeV infection from the apical surface, but infected cells were sloughed off and virus was unable to penetrate the intact epithelium (29). As for CDV, we found that cell-free CDV is unable to support a stable infection of canine airway epithelial cells, either from the basolateral or from the apical surface, even after removal of the mucus layer. Similarly, using macaque ALI cultures, only the edges of mechanically disrupted (scratch wound assay) cell layers became infected by CDV (27). In order to investigate the impact of epithelial disintegration on CDV infection, we treated ALI cultures prior to infection with EGTA. Pretreatment with this calcium chelator has been shown to disrupt the tight junctions of bovine ALI cultures and allow herpesviruses to infect epithelial cells via the paracellular route, targeting the receptor nectin-1 (30). Loss of epithelial junction integrity following EGTA treatment increased the CDV infection rate in ALI cultures. In addition, experiments with receptor-blind viruses indicate that usage of nectin-4 by CDV is related to the paracellular infection route. CDV-5804PeH and its mutant viruses were adapted to ferret cells by several passages *in vivo* (31); this adaptation process could partially explain why the replication of CDV-5804PeH in ferret ALI cultures was more efficient than that in cultures derived from dogs. Here, filters with pore sizes of 0.4 μm and 1.0 μm were applied to investigate the infectivity of cell-free CDV. The results show that direct infection of canine ALI cultures by CDV is limited and that the pore size does not affect the infection process. When the cultures were inoculated with CDV at an MOI of 0.5 (2.5 × 10^5^ 50% tissue culture infective dose [TCID_50_]), the average number of CDV foci was between 1.167 to 3.5 foci per well. However, when the canine ALI cultures grown on a 0.4-μm filter were inoculated with the same amount of cPIV5, which uses sialic acids as receptor determinants (32), the infection was successfully initiated from both the apical side (an average of 142.4 foci/well) and the basolateral side (an average of 188.8 foci/well) (Fig. S3). Different factors that inhibit cell-free CDV from infecting airway epithelium have to be considered.

Unlike studies of MeV infections in human or macaque ALI cultures, which are more efficient from the basolateral side, CDV infection of canine ALI cultures showed a significant increase in the number of infected cells when the virus was applied to the apical surface of EGTA-pretreated cells. A possible explanation is that the main epithelial receptor nectin-4 is situated midway up to the cell-to-cell junctions in the adherens junctions. From the apical perspective, the adherens junction is situated below the tight junction and hence is not accessible for the virus. Additionally, nectin-4 is arranged as a *trans*-homodimer with a canonical adhesive interface, which may veil the distal terminal V domain, the binding site recognized by the CDV hemagglutinin protein; this conformational restriction may limit virus attachment. For MeV, it has been shown that, if nectin-4 forms a strong binding between nectin-4 homodimers, infection by MeV is strongly reduced (33). The comparison of protein sequences of human and canine nectin-4 shows a glutamine-to-arginine substitution at position 78. Since this amino acid plays a key role in nectin-4 homodimer formation (34), the nectin-4 of dogs may have a different adhesive affinity than that of human nectin-4. Hence, we hypothesize that canine nectin-4 forms more closed types of *cis*- and *trans*-homodimers in primary canine airway epithelial cells, which block the access of CDV and thus prevent infection of primary cells from the basolateral side. When the cell-to-cell junctions have been interrupted via a scratch or EGTA treatment, the V domain of nectin-4 monomers becomes exposed, allowing CDV to attach to the receptor and successfully initiate the infection. In addition, our immunostaining results showed that the average nectin-4 exposure on the canine ALI culture was increased after EGTA pretreatment (see Fig. S4A to C in the supplemental material), which may partially explain why the interruption of tight/adhesion junctions affects the susceptibility of canine ALI cultures to CDV. Moreover, the binding affinity of the viral hemagglutinin (H) protein to the nectin-4 receptor may also be responsible for infectivity differences between CDV and MeV. Analyses of the MeV H protein three-dimensional (3D) structure reveal that the binding affinity of the H protein to the receptors is dominated by hydrophobic interactions (35). Based on the similarity of the hydrophobic pocket structure of CDV and MeV H proteins (20), together with the highly consensus FPAG motif present in canine and human nectin-4 (36), the binding affinity of CDV and MeV to nectin-4 may be similar (37, 38). It would be interesting to clarify the binding affinity between the H protein of CDV and receptors in future studies.

During the late stage of CDV infection, infected immune cells are thought to infiltrate the airway epithelium and transmit the virus to airway epithelial cells for releasing the virus via aerosols, as observed for MeV infection (23). *In vitro* studies revealed that MeV-infected human macrophages gain the ability to stretch and migrate through filter membranes with a pore size of 3.0 μm and to transmit infection to differentiated airway epithelial cells (26). Immunocyte-mediated transmission of MeV infection has also been demonstrated with 3D culture systems (39, 40). It is believed that after the virus is systemically disseminated in the body, immunocytes carry MeV back to the subepithelial region and transmit the infection to airway epithelial cells (41).

As shown in infection studies with ferrets, CDV dissemination and aerosol transmission is related to two distinct receptors, SLAM and nectin-4 (16). In contrast to the situation with MeV-infected ALI cultures, the results described here show a reduced ability of free CDV to infect the airway epithelium. To prove the hypothesis that immune cells transmit CDV to epithelial cells, an immunocyte-epithelial cell coculture model with the combination of canine ALI cultures and DH82 cells was established. Here, we tried two different permanent canine cell lines (MDCK and DH82) to propagate CDV-5804PeH-related viruses. However, CDV-5804PeH and SLAM-blind virus could infect MDCK cells, but not DH82 cells (see Fig. S2). For this reason, we used the CDV-OS virus for cell-to-cell transmission experiments. DH82 cells represent a permanent macrophage cell line isolated from a dog with histiocytic sarcoma and are commonly used for investigations of intracellular pathogens. The DH82 cells show phagocytic activity and produce macrophage-derived cytokines (e.g., interleukin 6 [IL-6], tumor necrosis factor [TNF]) and immune molecules involved in antigen recognition (e.g., CD14), antigen presentation (e.g., MHC class I), cellular adhesion (e.g., CD11c), and costimulation (e.g., CD86) (42–44). DH82 cells can migrate and show proteolytic activity (matrix metalloproteinase [MMP] expression) that enables extravasation (45, 46). Moreover, DH82 cells can show dendritic cell maturation upon stimulation (47). Previous studies showed that DH82 cells are susceptible to CDV-OS infection and are able to initiate virus production (48–50) and cell-associated virus transmission (51). Here, SLAM/F1 gene expression of DH82 cells was confirmed by real-time reverse transcription-quantitative PCR (qRT-PCR) (Fig. S5).

10.1128/mBio.03043-21.5 FIG S5 Gene expression of *SLAM/F1* and *PVRL4* (nectin-4) in canine cells. The gene expression of two CDV receptors was compared in MDCK, DH82, and canine primary tracheal epithelial cell (PTEC) cultures. Extracted total RNA was used to perform one-step reverse transcription-quantitative PCR (qRT-PCR). Detected threshold cycle (*C_T_*) values were normalized to MDCK cells. (A) *SLAM/F1* gene expression in DH82 cells was higher than that in MDCK and PTEC cultures. (B) *PVRL4* (nectin-4) was expressed at low levels in DH82 cells. Download FIG S5, TIF file, 0.3 MB. Copyright © 2022 Shin et al. 2022 Shin et al. https://creativecommons.org/licenses/by/4.0/ This content is distributed under the terms of the Creative Commons Attribution 4.0 International license.

When applied to the apical surface of canine ALI cultures, preinfected DH82 cells did not efficiently attach to the apical surface and were further removed by the mucous-ciliary clearance activity on day 3 post coculture. In contrast, applying preinfected cells to the basolateral surface of cocultures with a pore size of 1.0 μm resulted in virus infection of epithelial cells. Interestingly, no infection was found when canine ALI cultures with a pore size of 0.4 μm were used. Previously, it was shown that a pore size of 0.4 μm limits dendritic cells from direct contact with the opposite chamber, while a pore size of 1.0 μm was sufficient to stretch the cell podosome through the filter for antigen sampling (52). In our immunocyte-epithelial coculture system, DH82 cells presented protrusive structures, which facilitate virus transmission from DH82 cells to ALI cultures. In our experiments, a pore size of 0.4 μm was not enough for infected DH82 cells to pass through, whereas a pore size of 1.0 μm enabled virus transmission. Our results support the notion that cell-to-cell transmission may be a major route for CDV to infect airway epithelial cells. Cytoskeletal filamentous structures that extend from infected cells to neighboring cells may allow the exchange of viral components (53), as reported for some human paramyxoviruses (respiratory syncytial virus and human metapneumovirus) (54, 55). Whether this is also true for CDV has to be investigated in the future.

Previous studies of CDV-infected ferrets revealed that the virus is present in the trachea and lung region and is transmitted to other animals via aerosols (7). Other *in vitro* studies showed that MeV spreads by direct contact to neighboring epithelial cells rather than being released at the apical surface in human ALI cultures (25). Moreover, recent evidence had shown that, in the late stage of MeV infection, infected cells accumulate in the lumen and are removed and transmitted via the mucociliary clearance system of the airway epithelium (29, 56). As far as virus release is concerned, we got a result similar to that reported recently for MeV infection (29). When epithelial cells were infected by CDV, virus released into the supernatant was mainly present as cell-associated virus, i.e., within detached cells, and not as cell-free virus. Small amounts of infectious cell-free CDV have also been reported from studies using macaque ALI cultures (27).

Morbilliviruses have been shown to affect interferon-related innate immune responses, e.g., disturbing the JAK/STAT signaling pathway (9, 57). In our previous work, we revealed that inhibition of the JAK/STAT signal pathway in porcine ALI cultures by ruxolitinib mimics the situation of interferon stimulation failure of epithelial cells following influenza virus infection (58). Accordingly, while adapting the same methodology to canine ALI cultures, it was only when the cells were treated with ruxolitinib and cocultured with preinfected DH82 cells that infected epithelial cells showed increased syncytium formation, spread of viruses, and release of infectious virus particles at the apical surface. Similarly to MeV, CDV may use its V protein to interact with MDA5 and STAT2 to modulate the interferon signaling pathway (18). We showed here that restricting the type I interferon signaling cassette in the airway epithelium largely enhanced the cytopathic effect of CDV cell-to-cell transmission even with the vaccine strain. Here, we used CDV-OS with an intact V protein to perform the experiments. The CDV-OS strain had been used as a common live-attenuated vaccine, and hence the probability of transmission to another individual is likely to be negligible (59). A previous report showed that, due to a Y110D mutation in the V protein of CDV-OS, the ability of the V protein to inhibit the interferon induction cascade is limited (60). Nevertheless, the combination of ruxolitinib treatment with CDV-OS allowed us to mimic disturbed interferon signaling following CDV infection. Other CDV wild-type strains, such as Ohio R252 and A75/17 strains, may cause disease in experimentally infected dogs; however, these viruses are not available as recombinant viruses and have yet to be studied in this respect (61, 62). Similarly, CDV-5804PeH leads to lymphopenia and immunosuppression, but this strain is only able to infect ferrets (63). Further studies using different CDV wild-type viruses may help to clarify the influence of immune suppression on aerosol transmission and virus shedding.

CDV infection is used as an alternative model to study MeV-related pathogenesis. Both morbilliviruses show similarities but also slight differences in advanced ALI models. Here, we showed that, unlike MeV, which can infect ALI cultures from the basolateral side, CDV requires additional conditions to overcome the epithelial barrier of the airway epithelium, namely, (i) interruption of junctional complexes, which exposes nectin-4 and makes it accessible for infection by CDV during the paracellular route, (ii) immunocytes that carry CDV to facilitate cell-to-cell transmission; (and (iii) restricted interferon signaling that enhances virus release from the apical surface.

Further investigation is required to improve our insight into the dynamic interactions between the host organism and CDV.

## MATERIALS AND METHODS

### Viruses.

Recombinant CDV based on the Onderstepoort vaccine strain (CDV-OS) was generated as previously described (64); the CDV-OS contains an enhanced green fluorescent protein (eGFP) gene upstream of the nucleocapsid gene. Recombinant wild-type CDV-5804PeH was reverse-genetically modified from a ferret-adapted wild-type virus as previously described (31) and harbors the eGFP gene upstream of the polymerase (L) gene. Two mutant strains of CDV-5804PeH designated “nectin-4-blind” and “SLAM-blind” viruses contain mutations in the receptor-binding domain of the hemagglutinin protein (63, 65). All recombinant modified CDVs were propagated in Vero.dog-SLAMtag (VDS) cells for limited passages, and aliquots of viral titers of 5 × 10^6^ TCID_50_/mL were prepared.

### Immortalized cells.

The DH82 canine histiosarcoma-derived cell line was maintained in RPMI 1640 medium (Genaxxon, Ulm, Germany) with 12% fetal bovine serum (FBS) and showed a semiadherent phenotype. The VDS cells were generated as previously described (31). The VDS cells stably express a recombinant HA (influenza virus hemagglutinin-derived peptide)-tagged canine SLAM receptor and are maintained under the selective antibiotic pressure of Zeocin. VDS cells were cultured in Dulbecco’s modified Eagle’s medium (DMEM; Sigma-Aldrich) containing 5% FBS and 500 μg/mL of Zeocin (66). Madin-Darby canine kidney (MDCK) cells were maintained in DMEM medium containing 5% FBS and penicillin/streptomycin.

### Primary canine and ferret tracheal epithelial cells.

Canine tracheas were obtained from autopsy cases at the Department of Pathology of Veterinary Medicine, University Hannover, Germany. The protocol for isolating the canine primary tracheal epithelial cell (canine PTEC) was adapted from our previously established swine and ferret PTEC protocols (58, 67). In brief, detached epithelial cells from the mucosal parts of the epithelium were collected after protease XIV/DNase I incubation. Following removal of fibroblast cells by adherence to a petri dish, the PTECs were seeded in collagen-I-coated cell culture flasks (Nunc) at 37°C with 5% CO_2_ until they reached confluence. For infection experiments with ferret cells, cryopreserved ferret PTECs from previous studies were used (58). The growth medium for primary canine and ferret epithelial cells was based on the PneumaCult-Ex medium (Stemcell Technologies, Vancouver, Canada) with additional supplements, including 1 μM A83-01, 0.2 μM DMH-1, and 0.5 μM CHIR-99021 (Sigma-Aldrich) for increasing the cell vitality. The canine cells were tested by PCR to confirm that they were free of canine distemper virus, canine parainfluenza virus, canine coronavirus, and Bordetella bronchiseptica.

### Differentiation of airway epithelial cell cultures.

To establish well-differentiated canine ALI cultures, primary canine tracheal epithelial cells were transferred to Matrigel (Corning, United Kingdom)-coated filter supports with different pore sizes, including 0.4 μm (Sarstedt, Nümbrecht, Germany) and 1.0 μm (VWR International). Ferret PTECs were transferred to Matrigel (Corning)-coated filter supports with a pore size of 0.4 μm. PneumaCult ALI medium (Stemcell Technologies, Vancouver, Canada) was used during differentiation. After PTECs had reached confluence, the medium was removed from the apical surface, and cultures were maintained under air-liquid interface (ALI) conditions for at least 3 weeks at 37°C and 5% CO_2_ until cultures were well differentiated. In order to determine the polarization and barrier function of ALI cultures, we monitored the transepithelial electrical resistance values (TEER) changes during the whole differentiation process, as described previously (68). Semithin sections from well-differentiated ALI cultures were embedded in epoxy resin and analyzed by light field microscopy.

### Immunofluorescence analysis of well-differentiated ALI cultures.

The ALI cultures on filter supports were fixed with 3% paraformaldehyde (PFA) and permeabilized with 0.5% Triton X-100. The cultures were blocked with 1% bovine serum albumin (BSA) in phosphate-buffered saline with Tween 20 (PBST) buffer and incubated with primary antibodies, followed by incubation with fluorescence labeled secondary antibodies. The nuclei were counterstained with DAPI (4′,6-diamidino-2-phenylindole), and ALI cultures were embedded with ProLong Diamond mountant (Life Technologies) on microscope slides. The following antibodies were used: anti-mucin-5AC antibody (Acris), Cy3-labeled antibody against β-tubulin (Sigma-Aldrich), and secondary antibodies conjugated with Alexa Fluor 488 dye (Life Technologies). ALI cultures were analyzed with the Nikon Eclipse Ti microscope and the Leica TCS SP5 confocal microscope. NIS-Elements AR software 3.2 (Nikon), LAF AF lite software (Leica), and ImageJ/Fuji software (National Institutes of Health) were used for image processing. The experiments were repeated at least with six ALI cultures from three independent dogs (*n* ≥ 6).

### Immunohistochemical analysis of lung tissue from naturally CDV-infected dogs.

For immunohistochemistry, lung tissues obtained from necropsy cases were fixed in 4% formaldehyde solution for 24 h, routinely processed, and embedded in paraffin. Paraffin-embedded lung material from naturally CDV-infected dogs was then sectioned at 2 μm and immunohistochemically stained using the avidin-biotin-peroxidase complex method (69, 70). Infected lung samples were obtained from a juvenile, female, nonvaccinated mongrel dog. The dog showed neurologic and respiratory signs and was euthanized due to worsening of clinical symptoms. Main necropsy findings include demyelinating leukoencephalitis, depletion of lymphoid organs, and interstitial pneumonia.

In brief, lung sections were deparaffinized by Roticlear (Carl Roth) with subsequent rehydration through graded alcohols. Endogenous peroxidase activity was suppressed with H_2_O_2_ (0.5%) in 85% ethanol. Heat-induced antigen retrieval pretreatment was performed via incubation of the samples in citrate buffer (pH 6.0) for 20 min in a microwave (800 W). Unspecific bindings were blocked with goat normal serum (1:5) for 30 min. The primary antibody (mouse anti CDV-NP, clone D110, monoclonal; dilution, 1:1000) was incubated overnight at 4°C. For negative controls, the primary antibody was substituted with ascites fluid from nonimmunized BALB/c mice. The secondary antibody (biotinylated goat anti-mouse, polyclonal; dilution, 1:200; Vector Laboratories) was incubated for 45 min at room temperature. Subsequently, the avidin-biotin-complex (Vectastain Elite ABC kit; Vector Laboratories) was incubated for 20 min at room temperature. Antigen-antibody reactions were visualized by incubation with 3.3′-diaminobenzidine tetrahydrochloride (DAB) with H_2_O_2_ (0.03%) for 5 min, followed by counterstaining (30 s) with Mayer’s hemalum.

### Analyses of virus receptor expression in different canine cell cultures.

In order to determine whether canine cell cultures express the receptors for CDV, real-time qRT-PCR was performed. MDCK cells, DH82 cells, and primary canine PTECs were grown in 24-well plates. Approximately 5 × 10^5^ cells per well were collected, and total RNA was extracted using the RNeasy kit (Qiagen, Germany) according to the manufacturer’s instructions. Two microliters of RNA were used for detecting gene expressions of canine SLAM/F1 and PVRL4 (nectin-4) using the QuantiTect SYBR green RT-PCR kit (Qiagen). Canine β-actin was used as a housekeeping gene. Primers used for qRT-PCR are listed in Table S1 in the supplemental material. Real-time PCRs were performed using the Mx3005p real-time PCR system (Agilent). The PCR program included reverse transcription at 50°C for 30 min, followed by denaturation at 95°C for 3 min. PCR cycles included 95°C for 30 s (denaturation), 54°C for 30 s (annealing), and 72°C for 30 s (elongation). Finally, a melting curve analysis was performed. Data were statistically analyzed using the cycle threshold (ΔΔ*C_T_*) method. The MDCK group was selected as a normalization control.

10.1128/mBio.03043-21.6 TABLE S1 Primers used for one-step reverse transcription-quantitative PCR (qRT-PCR). Download Table S1, DOCX file, 0.03 MB. Copyright © 2022 Shin et al. 2022 Shin et al. https://creativecommons.org/licenses/by/4.0/ This content is distributed under the terms of the Creative Commons Attribution 4.0 International license.

### Canine distemper virus infection of ALI cultures and EGTA pretreatment.

Prior to CDV inoculation on ALI cultures, well-differentiated ALI cultures were washed five times with sterile PBS buffer. In total, 50 μL of DMEM medium containing CDV with 2.5 × 10^5^ TCID_50_ (MOI of 0.5) were applied on the apical or basolateral side in an upside-down position for 2 h of incubation time at 37°C. The fluorescence signal from virus expressing eGFP was monitored daily using the Nikon Ti microscope. Apically released virus was collected by inoculating the cells with 100 μL DMEM for 30 min (67), and virus released from the basolateral side was directly harvested by collecting medium in the lower chamber (67). TCID_50_ tests were directly performed by adding the samples to confluent VDS cells in 24-well plates. Formation of cell syncytia on VDS cells proved viral replication and was used to determine the virus titer after 3 days. To allow the virus to enter the airway epithelial cells via the paracellular route, ALI cultures were pretreated with EGTA (Sigma-Aldrich) prior to CDV infection. EGTA 0.1 M in PBS without calcium and magnesium was directly applied to both the upper and lower chambers of ALI cultures and incubated at 37°C for 10 min. Following this pretreatment, cells were washed immediately with PBS to remove remaining EGTA, and the cell-free virion inoculation was started as described previously (30).

### Establishing an immunocyte-epithelial transwell coculture system.

In order to prepare CDV-infected DH82 cells, 2 × 10^4^ DH82 cells were seeded in 12-well plates in DMEM containing CDV-OS (MOI of 0.1) at 37°C. On the next day, the medium was discarded, and cells were supplied with fresh DMEM containing 2% FBS. CDV-OS-infected DH82 cells were kept at 37°C with 5% CO_2_ for an additional 48 h prior to coculture experiments. For coculturing, CDV-OS-preinfected DH82 cells were detached gently and collected in DMEM. After mucus was removed by five washes with PBS, 200 CDV-OS-infected DH82 cells were applied to the apical surface or to the basolateral side in the upside-down position of ALI cultures for 4 h at 37°C. The washing procedure resulted in only a minor reduction of the TEER and did not abolish the epithelial integrity (Fig. S4D). After incubation, nonadhered DH82 cells were washed away gently by PBS, and ALI cultures were continuously cultured under previous conditions. On the next day, one drop (33 μL) of 1% Matrigel in ice-cold DMEM/F-12 (Gibco) was applied to the basolateral side of the filter support to avoid the detachment of basolaterally adhered DH82 cells and to maintain the coculture condition of DH82/ALI cells. DH82/canine ALI cocultures were monitored for 6 days.

### JAK/STAT signaling pathway inhibition.

For inhibiting the downstream stimulation of interferon related pathways, ruxolitinib, a potent JAK/STAT signaling pathway inhibitor, was applied in ALI cultures and DH82/ALI cocultures. Three days prior to either cell-free virion inoculation or coculturing, ALI cultures received ALI medium containing 2.5 μM ruxolitinib (Invivogen, France) as previously described (58). During cell-free virion inoculation or coculture experiments, ruxolitinib was continuously supplied in the ALI medium. Ruxolitinib-treated and nontreated cells were monitored for 6 days for eGFP signals using the Nikon Eclipse Ti microscope. Released virus particles were collected, and virus titers were determined as described above.

### Statistical analysis.

Data processing and statistical analysis were performed using Prism version 8.01 (GraphPad Software, California). Results are presented as mean ± standard error of the mean (SEM) for the quantity of virus foci and as TEER value changes. Statistically significant differences between groups were determined using the one-way analysis of variance (ANOVA) test with Tukey’s *post hoc* analysis.
