# Supplementary figures and images for: Overcoming the Barrier of the Respiratory Epithelium during Canine Distemper Virus Infection
Source: mBio. 2022 Jan 18;13(1):e03043-21. doi: 10.1128/mbio.03043-21 (PMC8764546; doi:10.1128/mbio.03043-21)

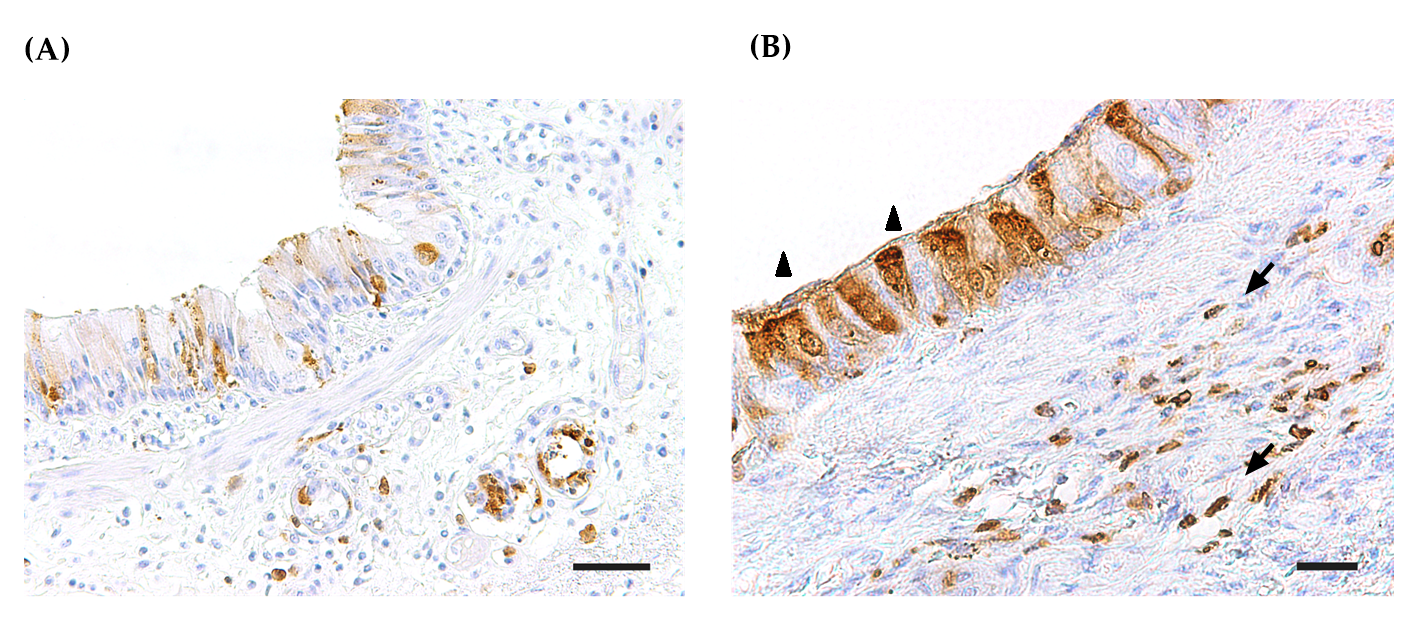

Supplement: FIG S1 [file mbio.03043-21-sf001.tif]

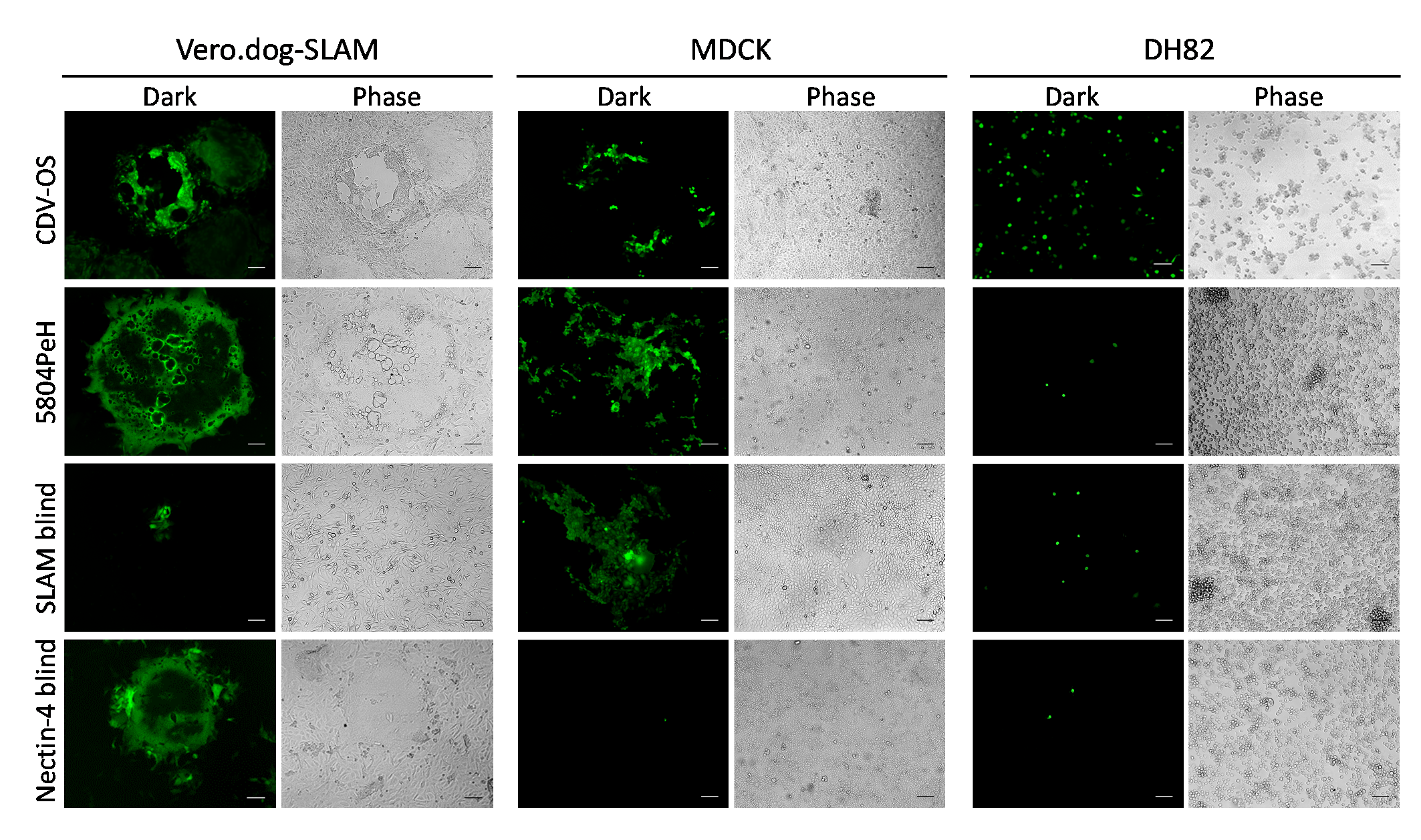

Supplement: FIG S2 [file mbio.03043-21-sf002.tif]

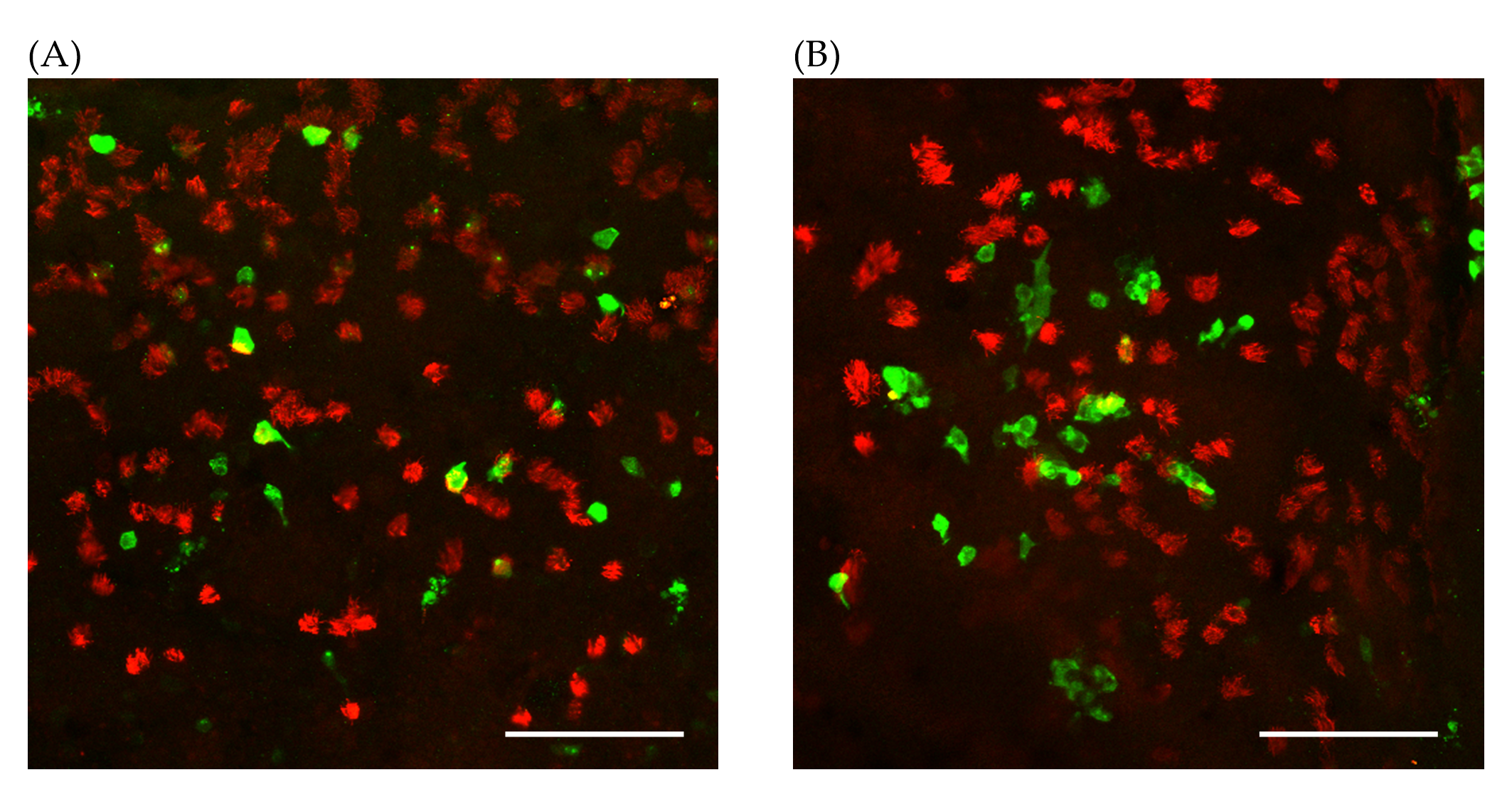

Supplement: FIG S3 [file mbio.03043-21-sf003.tif]

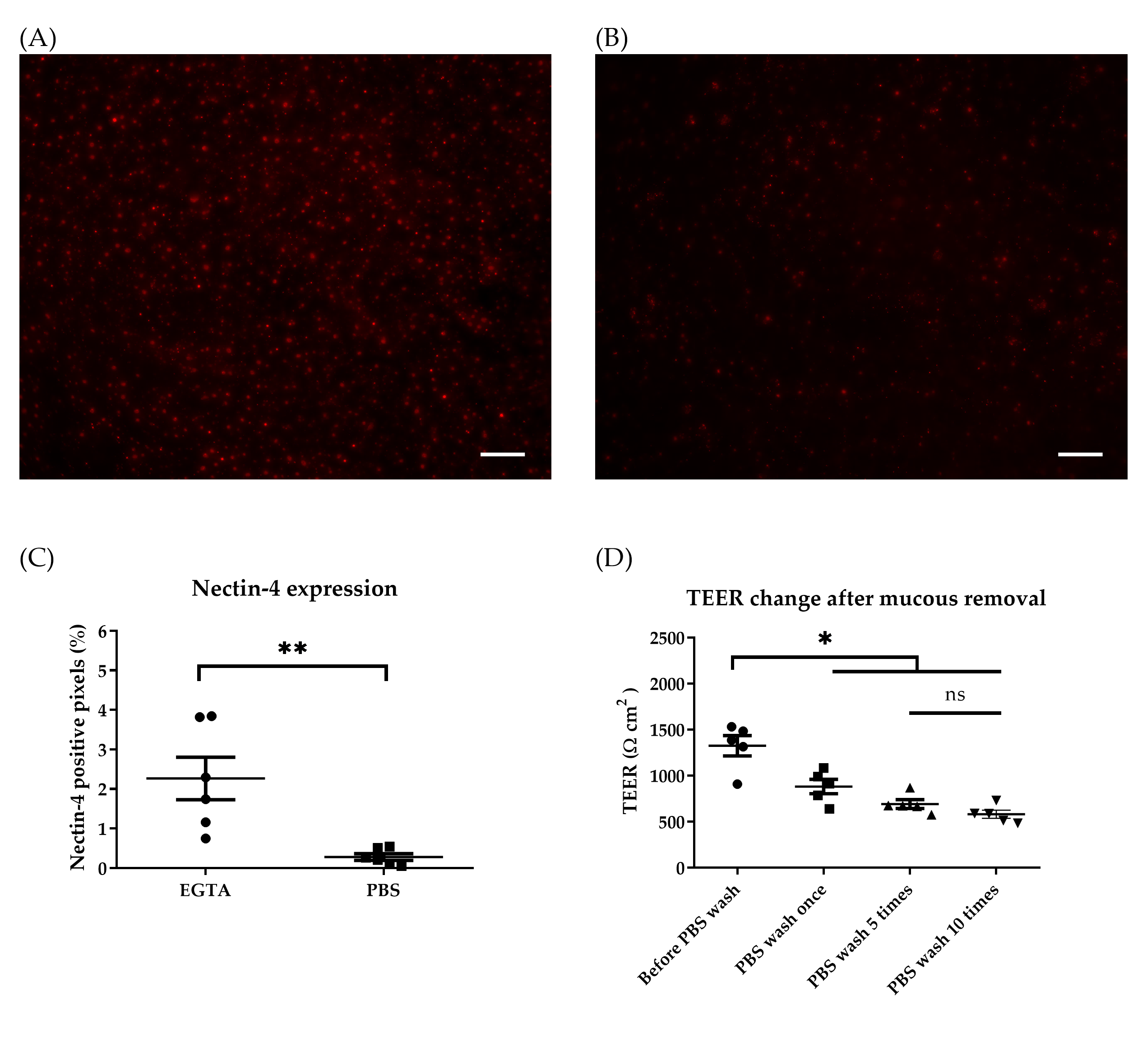

Supplement: FIG S4 [file mbio.03043-21-sf004.tif]

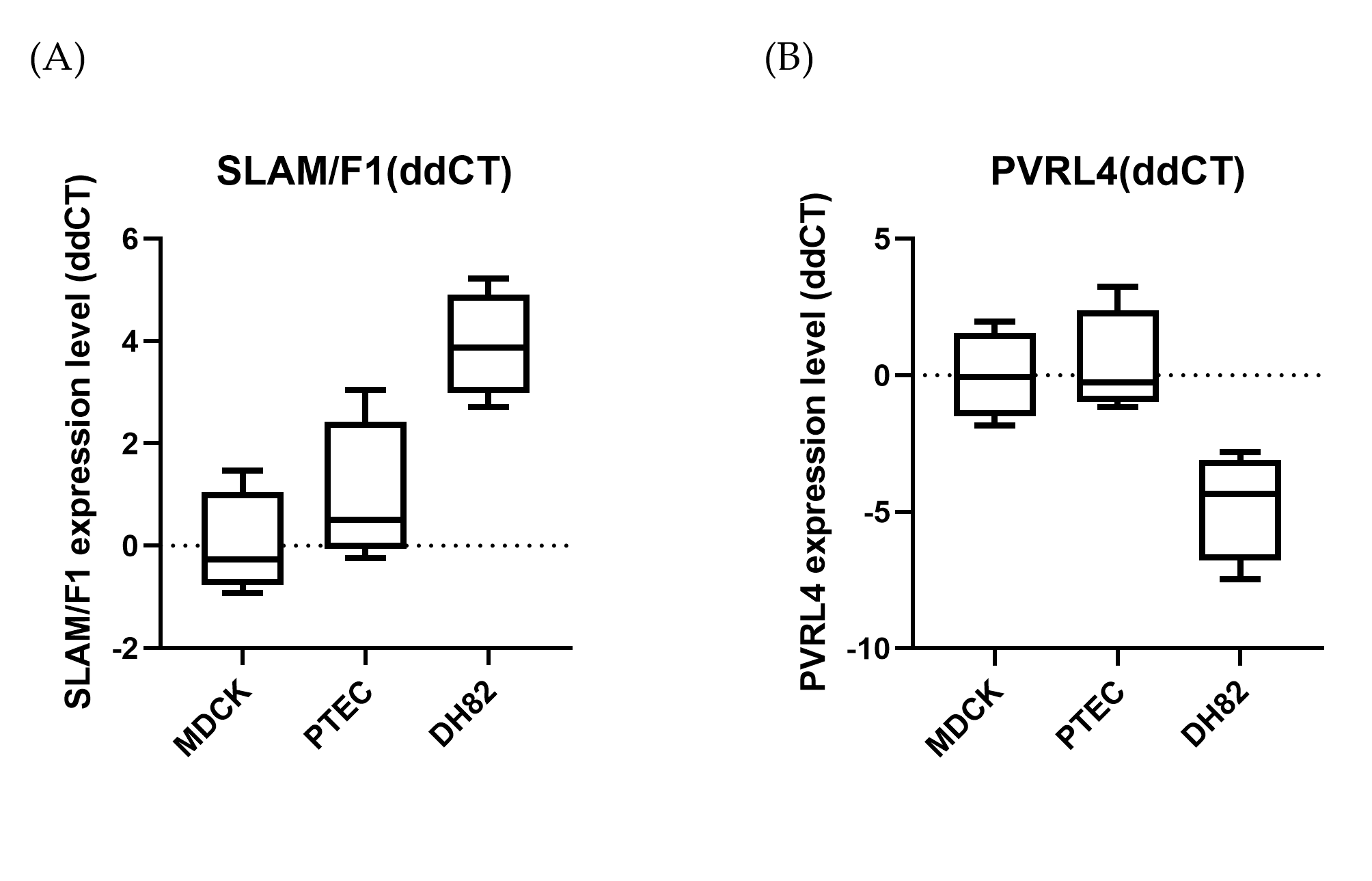

Supplement: FIG S5 [file mbio.03043-21-sf005.tif]
