# Supplementary material for: Overcoming the Barrier of the Respiratory Epithelium during Canine Distemper Virus Infection
Source: mBio. 2022 Jan 18;13(1):e03043-21. doi: 10.1128/mbio.03043-21 (PMC8764546; doi:10.1128/mbio.03043-21)
Supplement: TABLE S1 [file mbio.03043-21-st001.docx]

Table S1. Primers used for one step qRT-PCR.

| Primer | Target | Sequence | Reference |
| --- | --- | --- | --- |
| cSLAM-F | Canine *SLAM/F1* | GCCAAGACAATGGAGACT | This study |
| cSLAM-R | Canine *SLAM/F1* | ATCAAGATGACACCAACGAT | This study |
| cPVRL4-F | Canine *Nectin-4* | GTGGTGGTGCTCATGTC | This study |
| cPVRL4-R | Canine *Nectin-4* | CAGCTCCTCCTCGTATT | This study |
| cACTb-F | Canine *β-Actin* | CAAAGCCAACCGTGAGAAG | (1) |
| cACTb-R | Canine *β-Actin* | CAGAGTCCATGACAATACCAG | (1) |

Reference:

S1. Mak GZ, Kavanaugh GM, Buschmann MM, Stickley SM, Koch M, Goss KH, Waechter H, Zuk A, Matlin KS. 2006. Regulated synthesis and functions of laminin 5 in polarized madin-darby canine kidney epithelial cells. Mol Biol Cell 17:3664-77.
